# Sodium Arsenite-Induced Learning and Memory Impairment Is Associated with Endoplasmic Reticulum Stress-Mediated Apoptosis in Rat Hippocampus

**DOI:** 10.3389/fnmol.2017.00286

**Published:** 2017-09-07

**Authors:** Hongna Sun, Yanmei Yang, Hanwen Shao, Weiwei Sun, Muyu Gu, Hui Wang, Lixin Jiang, Lisha Qu, Dianjun Sun, Yanhui Gao

**Affiliations:** ^1^Key Lab of Etiology and Epidemiology, Education Bureau of Heilongjiang Province & Ministry of Health, Center for Endemic Disease Control, Chinese Center for Disease Control and Prevention, Harbin Medical University Harbin, China; ^2^Institution of Environmentally Related Diseases, Harbin Medical University Harbin, China

**Keywords:** arsenic, apoptosis, homocysteine, endoplasmic reticulum stress, hippocampus, blood brain barrier

## Abstract

Chronic arsenic exposure has been associated to cognitive deficits. However, mechanisms remain unknown. The present study investigated the neurotoxic effects of sodium arsenite in drinking water over different dosages and time periods. Based on results from the Morris water maze (MWM) and morphological analysis, an exposure to sodium arsenite could induce neuronal damage in the hippocampus, reduce learning ability, and accelerate memory impairment. Sodium arsenite significantly increased homocysteine levels in serum and brain. Moreover, sodium arsenite triggered unfolded protein response (UPR), leading to the phosphorylation of RNA-regulated protein kinase-like ER kinase (PERK) and eukaryotic translation initiation factor 2 subunit α (eIF2α), and the induction of activating transcription factor 4 (ATF4). Arsenite exposure also stimulated the expression of the endoplasmic reticulum (ER) stress markers, glucose-regulated protein 78 (GRP78), C/EBP homologous protein (CHOP) and the cleavage of caspase-12. Furthermore, exposure to arsenite enhanced apoptosis as demonstrated by expression of caspase-3 and TUNEL assay in the hippocampus. The results suggest that exposure to arsenite can significantly decrease learning ability and accelerate memory impairment. Potential mechanisms are related to enhancement of homocysteine and ER stress-induced apoptosis in the hippocampus.

## Introduction

Arsenic is a poisonous metalloid element in the environment. This element exists in water, air and soil with the form of inorganic arsenic (iAs). Two main species of iAs are found in ground water: arsenite (the trivalent state, As^III^) and arsenate (the oxidized pentavalent state, As^V^). Millions of people drink water containing high levels of arsenic in the countries such as Mexico, Bangladesh, China and India (Nordstrom, 2002; Sengupta et al., 2008). Chronic exposure to arsenic via drinking water has been connected with numerous cancers (e.g., skin, lung and bladder) and non-cancer health conditions (Oberoi et al., 2014; Steinmaus et al., 2014; Tolins et al., 2014). Recently, the neurotoxic effect of arsenic has attracted scientific attention.

Epidemiological and clinical studies have indicated that people exposed to arsenic exhibit serious central nervous system injury such as cognitive dysfunction, behavioral deficits and mood disorders (Mathew et al., 2010; Liu et al., 2017). Long-term exposure to arsenic induced changes that to be in line with most of the pathologic, clinical and developmental features of Alzheimer disease (AD) and associated disorders (Gong and O’Bryant, 2010). Children are particularly sensitive to arsenic induced neurotoxicity (Roy et al., 2011). Animal experiments have found that arsenic induced brain damage altered the normal behaviors and impaired neuro development neural morphology, neuroinflammation and induced apoptosis (Yen et al., 2011; Flora et al., 2012). Furthermore, studies have shown that arsenic could induce toxicity in HAPI microglia (Mao et al., 2016), cerebellar granule neurons (Liu et al., 2013) and central snail neurons (Lu et al., 2009). Altogether, arsenic can lead to neuronal cell death, but detailed mechanisms of arsenic-induced neurotoxicity are not completely understood.

Among mechanisms underlying the neurotoxic effect of arsenic, apoptosis has been proved to play an important role in the arsenic-induced neurodegeneration. There is increasing evidences showed that arsenic could induce cell apoptosis by activating the endoplasmic reticulum (ER) stress pathway in diverse types of cells. Studies have shown that arsenic triggered the activation of glucose-regulated protein 78 (GRP78) and induced ER stress in osteoblasts (Tang et al., 2009) and renal cells (Kimura et al., 2005), and arsenic could phosphorylate the α subunit of eukaryotic translation initiation factor 2 subunit α (eIF2α) and induce the ER stress-mediated apoptosis in laryngeal squamous cells (Yang et al., 2014) and myoblasts (Yen et al., 2012). Studies also found that arsenic-induced apoptosis of the breast cancer cells (Zhang et al., 2015) occurred mainly by activating the ER stress associated proteins including C/EBP homologous protein (CHOP) and activating transcription factor 4 (ATF4).

Various elements can disrupt ER functioning and lead to the accumulation of misfolded proteins, thereby inducing ER stress (Weng et al., 2014). The ER stress triggers a signaling pathway involving RNA-regulated protein kinase-like ER kinase (PERK) and eIF2α kinase that phosphorylates the α subunit of eIF2, leading to the protein synthesis was restrained (Gao et al., 2013). Phosphorylated eIF2α selectively increases translation of ATF4, which may induce expression of unfolded protein response (UPR) target genes, involved in the ER-induced apoptosis (Weng et al., 2014). PERK, which has an endonuclease activity, may activate the expression of ER marker protein including GRP78. The GRP78 could prevent protein aggregation and contribute to protein folding. Subsequently, overmuch or continuous ER stress may induce ER-mediated apoptosis by activating the CHOP and caspase-12 (Boyce and Yuan, 2006; Gao et al., 2013). Although studies suggest that arsenic could induce neurotoxic effects in mammals, mechanisms underlying the neurotoxicity from arsenite induced ER stress have still not been well studied, especially in the hippocampus.

Previously, it has been reported that a high prevalence of hyperhomocysteinemia was found in the high arsenic water areas in Bangladesh (Yi et al., 2000; Gamble et al., 2005).

Homocysteine that, functions as a domain metabolic intermediate in sulfur-containing amino acid metabolism was generated from the metabolism of methionine (Williams and Schalinske, 2010). There is a general consensus that exposure to homocysteine induces death in various neuronal cell-types such as SH-SY5Y cells (Park et al., 2013), *in vitro* cultured cerebellar granule cells (Kuszczyk et al., 2009), *in vitro* cultured the Purkinje cells (Oldreive and Doherty, 2007) and rat cortical and hippocampal neurons (Abushik et al., 2014). Studies have also shown that homocysteine could not only change the hippocampal nerve toxicity but also alter the susceptibility to oxidative damage, which further resulted in deficiencies in learning and memory (Wang et al., 2012). Certain potential mechanisms have been discovered to explain the relationship between homocysteine and neurotoxicity, including oxidative stress (Ataie et al., 2010), tau protein phosphorylation (Zhang et al., 2008; Sontag et al., 2014), protein phosphatase 2A methylation (Sontag et al., 2014), immune activation (Boldyrev et al., 2013) and blockage of the nitric oxide signaling pathway (Jara-Prado et al., 2003). The ER stress is one of vital mechanisms (Althausen and Paschen, 2000; Kim et al., 2008; Wei et al., 2014). Homocysteine could trigger ER stress by influencing disulfide linkage and lead to UPR. *In vitro* homocysteine-induced ER stress can induce neuronal apoptosis (Kim et al., 2008). These results suggest that arsenic may injure the nerve system by enhancing the homocysteine level. However, until now, no study has researched the effect of homocysteine in the arsenic-induced neurotoxicity.

In this study, arsenic levels in serum and brain tissues were measured. The Morris water maze (MWM) was used to investigate the effect of arsenite on spatial learning and memory. Alterations in homocysteine levels and hippocampal morphology, induction of ER stress, and production of apoptosis in the hippocampus were detected. This is helpful for further understanding the mechanisms of sodium arsenite-induced impairment in learning and memory.

## Materials and Methods

### Animal Preparation and Study Design

**Figure 1:**
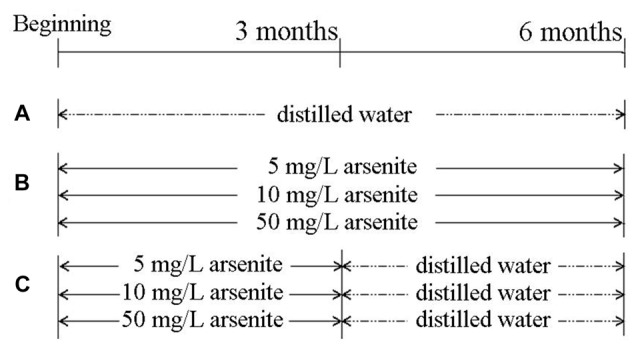
The scheme of the experimental group. **(A)** Control group. **(B)** Three groups, in which rats received 5, 10, 50 mg/L sodium arsenite six consecutive months. **(C)** Other three groups, in which rats received 5, 10, 50 mg/L sodium arsenite for 3 months and then received to distilled water for another 3 months.

Seventy 6-week-old male SD rats from the Vital River Laboratory Animal Technology Co. Ltd (Beijing) were used. Rats had common feedstuff and drank water freely and were kept at 20 ± 2°C and 55 ± 5% in relative humidity. After adaption for 1 week, rats were randomly divided into seven groups of 10 rats. Sodium arsenite was purchased from Reagent No. 2 Factory of Shanghai Chemical Reagent Co.Ltd (Shanghai, China). The control group received distilled water for 6 months. Three groups received 5, 10, 50 mg/L sodium arsenite for six consecutive months, and the other three groups received 5, 10, 50 mg/L sodium arsenite for 3 months and then received distilled water for another 3 months (Figure 1). Our pilot study provided an appropriate drug for this study (Zhang et al., 2013; Qu et al., 2016). All rat experiments were in strict accordance with the Chinese legislation on the use and care of laboratory animals and were approved by the University committees for animal experiments.

After arsenite exposure, 10 rats from each group were tested in the MWM. Then, rats were intraperitoneally anesthetized with 10% chloral hydrate. The whole blood samples were collected from femoral artery and were centrifuged at 3000 rpm for 10 min and serum was retained. The right hemisphere of eight rats was immersed in the paraformaldehyde medium. The right-side hippocampus of the last two rats in each group was quickly separated and fixed in glutaraldehyde, and then used for transmission electron microscopic (TEM) study. The left-side hippocampus was rapidly frozen in liquid nitrogen and then stored at −80°C.

### Total Arsenic Contents in Serum and Brain of Rats

The total arsenic contents in the serum and brain of rats were tested using hydride generation atomic fluorescence spectrometry, operate according to a national standardized method in China (GB/T 5009.11-2003). Two-hundred microlitres of serum or 0.2 g of brain samples were digested with 2 ml of concentrated nitric acid and 1 mL of perchlorate in the digestion system. Before measurement, 1 mL of 100 g/L thiourea ascorbic acid and 1 mL of concentrated hydrochloric acid was added to digested samples, then diluted to 10 mL. A blank sample was performed in the same way. Standard solution was prepared with standard reference materials (GBW08611). A hydride atomic fluorescence spectrometer (AFS-930, Beijing Titan Instruments, China) was used for detecting arsenic total levels. The calibration range of arsenic was from 0 mg/L to 100 mg/L.

### The MWM Test

The experimental apparatus consisted of a 120 cm diameter, 60 cm height circular water tank. The temperature of the water was kept at 24 ± 1°C. A 10 cm diameter, 35 cm height black escape platform was placed at one of four pool quadrants and submerged approximately 1 cm below the water surface. Each rat was trained four times for five continue days. Escape latency to find the submerged escape platform in the water maze were recorded. Each training session lasted for a maximum of 60 s. If escape latency exceeded 60 s it was recorded as 60 s and the rat was manually guided to the platform. On Day 6, the platform was removed and each rat swam freely for 60 s. The behavior of the animals was recorded with a video tracking system (SONY Inc., Japan) placed appropriately above the maze apparatus and analyzed by SMART 2.5, a video tracking software for behavioral experiments.

### Determination of Homocysteine Levels in Serum and Brain of Rats

The serum homocysteine level was measured by ARCHITECT Homocysteine Reagent Kit (Axis-Shield Diagnostics Ltd., Dundee, UK). The assay is a chemiluminescent microparticle immunoassay (CMIA) for the quantitative determination of total L-homocysteine in serum on the ARCHITECT i System. The measurement range of this assay is from 1.00 μmol/L to 50.00 μmol/L. Homocysteine in the rat hippocampus was measured by an ELISA kit from CusaBio Biotech Corp. Ltd. (CSB-E13376r), operated according to the manufacturer’s instructions.

### Hematoxylin and Eosin (H&E) Staining

The paraformaldehyde-fixed brain tissues were embedded in paraffin and cut into 4 μm thickness using a section cutter. The paraffin-embedded sections were cleared in xylene, gradually rehydrated in ethanol, and stained with H&E. The pathological changes of CA1 area of hippocampus were observed with Olympus Type BX53 microscopy.

### Transmission Electron Microscope (TEM)

About 1 mm^3^ tissue sample in CA1 area of the right-side hippocampus was separated and fixed in 2.5% glutaraldehyde at 4°C for 24 h and then rinsed in 0.1 M phosphate-buffered saline (PBS; pH 7.2–7.4). The tissues were fixed with 1% osmium tetroxide for 2 h, dehydrated in a graded series of acetone and embedded in resinene. Then thin slices were cut and stained with 4% uranyl acetate and 0.5% lead citrate. The ultrastructure of the CA1 area of hippocampus was observed with the transmission electron microscopy (H-600, Hitachi, Japan).

### Terminal Deoxynucleotidyl Transferase dUTP Nick End-Labeling (TUNEL) Assay

The TUNEL assay was performed on 4-μm thick paraffin-embedded sections using the Cell Death Detection Kit (Roche Applied Science, Germany). Tissue sections were deparaffinized in xylene, rehydrated, and immersed in 3% H_2_O_2_. Then, sections were treated with proteinase K solution on ice for 5 min and further incubated with 50 μl of TUNEL reaction mixture at 37°C for 60 min, and followed by being incubated with 50 μl of POD at 37°C for 30 min. These sections were stained by DAB and then stained by hematoxylin. Eight Slides were randomly chosen from each group and observed with light microscope. Three visual fields (200×) from each slide were randomly chosen in the CA1 region of hippocampus. The TUNEL-positive cells rate was obtained under Olympus Type BX53 microscopy.

### Immunohistochemistry

Immunohistochemistry was performed to measure GRP78 and CHOP expression. The brain paraffin slices were passed through xylene and ethanol and then blocked using 0.3% hydrogen peroxide solution for 15 min. Slices were subjected to microwave antigen retrieval with citric acid buffer (PH6.0) and blocked with 5% BSA for 2 h. Then the slices incubated with GRP78 antibody (1:2000, Cell Signaling Technology, Inc., Beverly, MA, USA) and CHOP antibody (1:1000, Santa Cruz Biotechnology, Santa Cruz, CA, USA) overnight at 4°C. Next, slices were incubated with avidin-biotin horseradish peroxidase complex (Boster Biotechnology, China) for 30 min at 37°C. To develop color, slices were incubated in a DAB staining kit (Boster Biotechnology, China) and counterstained with hematoxylin. The positive cells were stained brown under light microscope. The fields (200×) of the hippocampus CA1 region of eight sections were analyzed. The expression of GRP78 and CHOP were measured by ImageJ software by observing the density of immunopositive neurons.

### Immunoblot Analysis

Dissected hippocampal tissues were homogenized in RIPA buffer (Beyotime Institute of Biotechnology, China) containing protease and phosphatase inhibitors (Roche, Nutley, NJ, USA), using a micro-homogenizing system. The tissue homogenates were then centrifuged at 12,000 *g* for 10 min at 4°C, and the supernatants were collected as total protein extracts. The concentration of total protein was measured by the Bradford assay (Beyotime Institute of Biotechnology, China). Protein extracted from the hippocampal homogenate was mixed with sample loading buffer (Tris/HCl 300 mM, SDS 10%, Glycerol 50%, DTT 25 mM, Bromophenol Blue 0.05%) in a ratio of 4: 1 and boiled for 5 min. Protein extracts were subjected to electrophoresis on 10% (W/V) SDS–polyacrylamide gels then transferred onto a polyvinylidene fluoride membrane (Millipore, Bedford, MA, USA) by electrophoretic transfer. The membranes were incubated in blocking buffer (5% BSA prepared in Tris-buffered saline containing 0.1% Tween-20) for 1 h at room temperature and incubated overnight at 4°C in blocking buffer containing primary antibodies. The following primary antibodies and dilutions were used: GRP78 (1:1000), Caspase-12 (1:500; Abcam, Cambridge, UK); p-PERK (1:150), p-eIF2α (1:500), PERK (1:500), eIF2α (1:500), Caspase-3 (1:300; Cell Signaling Technology, Inc., Beverly, MA, USA); CHOP (1:1000, Santa Cruz Biotechnology, Santa Cruz, CA, USA); GAPDH (1:1000, Kangchen Biotechnology, China). Next, the membranes were incubated for 1 h with Goat anti rabbit IgG antibody conjugated to alkaline phosphatase (1:1000 dilution; Zhongshan Golden Bridge Biotechnology, China) as the second antibody. The band was visualized after reacting with Western Blue Stabilized Substrate for Alkaline Phosphatase (Promega, Madison, WI, USA) for a moment and was photographed by Tanon GIS-2020 gel image processing system (Shanghai Tanon Technology, China; Qu et al., 2016). The results were expressed as a relative optical density and analyzed using ImageJ software. Values based on five independent experiments were used for statistical analysis.

### Statistical Analysis

When compared with the control, significance was evaluated according to one-way analysis of variance (ANOVA) followed by Student-Newman-Keuls or Tukey’s Honestly Significant Difference *post hoc* test. When the data in arsenite for 3 months and water for 3 months group were compared with the corresponding 6 months group, the significance of difference was evaluated by the Student’s *t* test. Group differences in the escape latency in the MWM test were analyzed using two-way ANOVA with repeated measures, the factors being treatment and training day. Data are presented as means ± SD. Correlation analysis was used to assess the relationships between double quantitative variables. Partial correlation analysis was conducted to analyze correlations between arsenic in the brain and expression of GRP78, CHOP. The *P* value less than 0.05 was considered as significant. Statistical analysis was performed using SPSS19.0.

## Results

### Arsenic Contents in Serum and Brain of Rats

**Figure 2:**
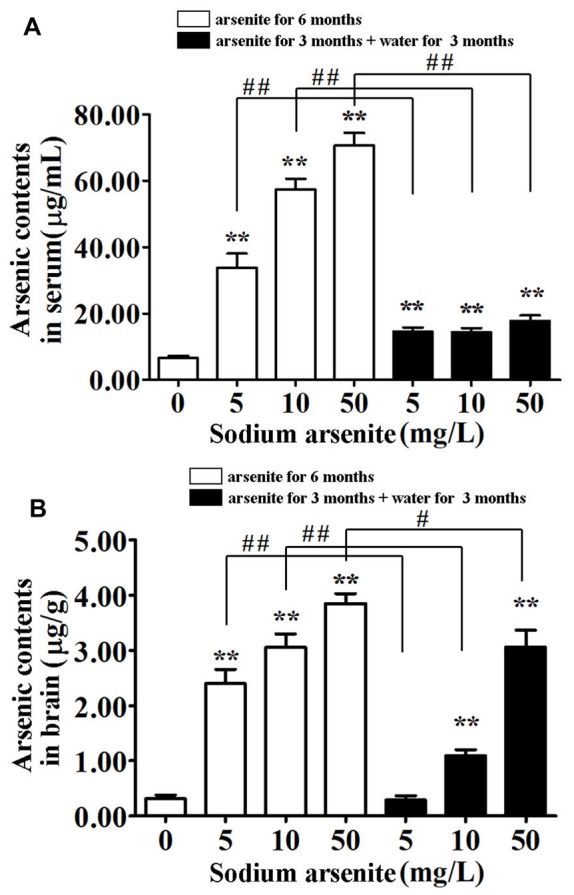
Arsenic contents in serum and brain of rats. Results represent mean ± SD and express in μg arsenic/ml serum **(A)** and μg arsenic/g brain **(B)**, respectively. *n* = 10. **P < 0.01 vs. control group. ^#^*P* < 0.05, ^##^*P* < 0.01.

In order to detect the accumulation of arsenic in rats, the arsenic contents in serum and brain were measured (Figure 2). The total arsenic contents in rat serum after daily exposure to arsenite for 6 consecutive months was significantly elevated as compared to the control. The contents reached up to 70.68 ± 12.03 μg/mL in the 50 mg/L groups, above 10 times higher compared to 6.47 ± 2.23 μg/mL in the control (*P* < 0.01). In the three groups of rats that were given arsenite for 3 months and distilled water for another 3 months, the serum arsenic levels were decreased significantly compared with the corresponding 6 months group (*P* < 0.01) and maintained under 20 μg/mL (Figure 2A). This result suggested that arsenic can be accumulated in the blood and excreted after halting arsenite exposure, but it can’t be completely excreted in a short time.

Brain arsenic levels increased with the arsenite exposure. The contents have already reached 3.85 ± 0.55 μg/g in the 50 mg/L group, compared to 0.31 ± 0.20 μg/g in the control (*P* < 0.01). Interestingly, after withdrawal period, brain arsenic levels of the 10 mg/L and 50 mg/L groups were still relatively high (1.10 ± 0.32 and 3.06 ± 0.96, respectively; Figure 2B). It was presumed that arsenic can pass through the blood-brain barrier (BBB) and accumulate in the brain, even after halting arsenite exposure.

### The MWM Test

**Figure 3:**
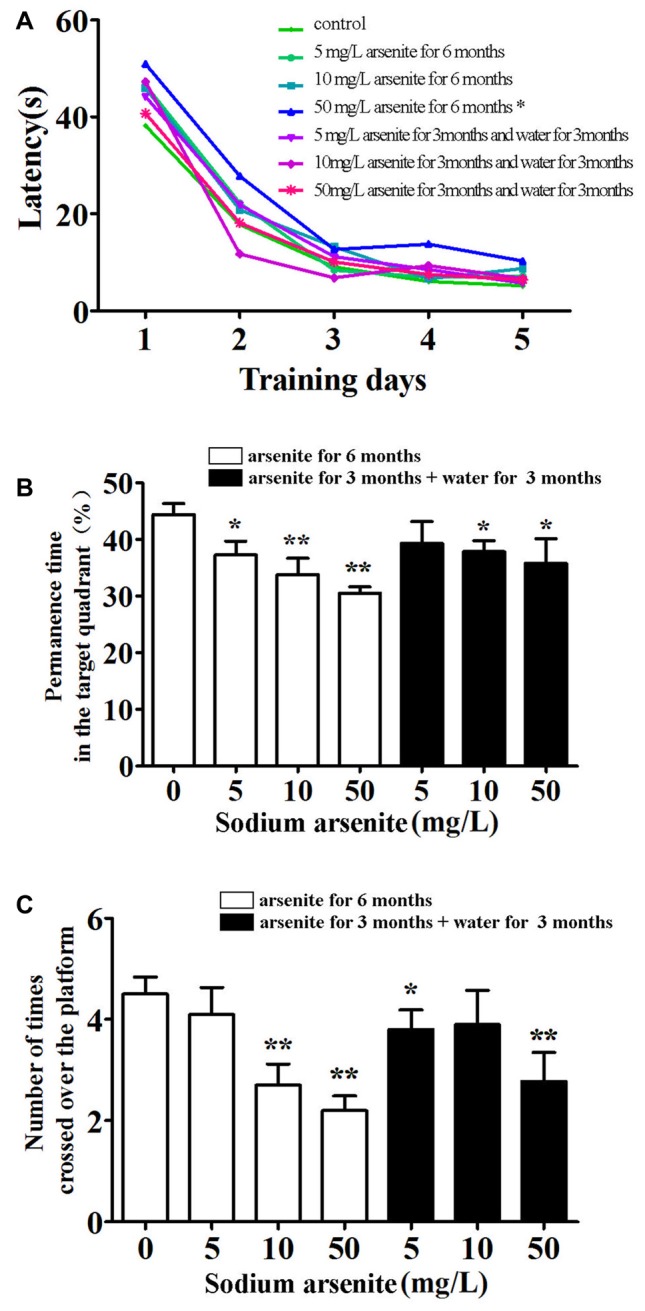
Morris water maze (MWM) test. **(A)** Mean latency in the spatial navigation test for first 5 days. **(B)** The percentage of time spent in target quadrant (where the platform was located during hidden platform training) on Day 6. **(C)** The number of crossings over the exact location of the former platform on Day 6. *n* = 10. *P < 0.05, **P < 0.01 vs. control group.

The MWM was conducted to investigate the spatial learning and memory of rats (Figure 3). Throughout 5 days of training, there was a significant difference in mean latency between training days and between treatments, but there was no interaction between the factors day and treatment. The results showed that only rats in the 50 mg/L arsenite-treated group for 6 months had longer escape latency compared with the control (*P* < 0.01; Figure 3A). Rats required longer time to find the escape platform suggesting a decline in spatial learning ability caused by arsenite. In the probe test on Day 6, the percentage of time, rats spent in target quadrant, was 44.34 ± 6.21%, 37.22 ± 7.83%, 33.83 ± 8.94% and 30.48 ± 3.73%, respectively in the control and arsenite-treated group for 6 months, which indicated that rats treated with arsenite spent less time than the control (*P* < 0.05 or *P* < 0.01). Similarly, rats in the arsenite-treated group for 6 months crossed over the platform less frequently than control rats (*P* < 0.05 or *P* < 0.01; Figure 3B). These data suggest that arsenite exposure could reduce learning and memory abilities.

### Homocysteine Levels in Serum and Brain of Rats

**Figure 4:**
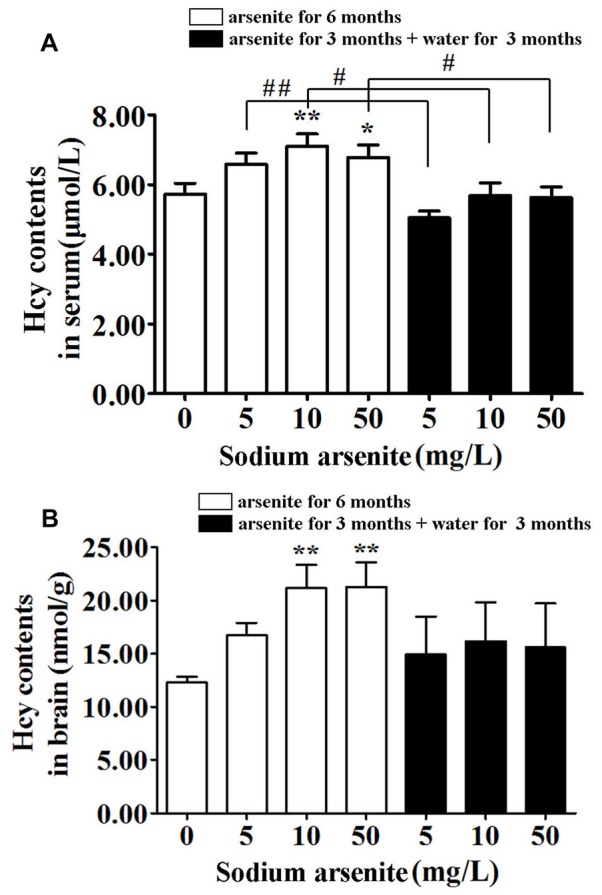
Homocysteine levels in serum and brain of rats. Results represent mean ± SD and express in μmol homocysteine/L serum (*n* = 10) **(A)** and μmol homocysteine/g brain (*n* = 5) **(B)**, respectively. *P < 0.05, **P < 0.01 vs. control group. ^#^*P* < 0.05, ^##^*P* < 0.01.

Previous research had found that a prevalence of hyperhomocysteinemia was high in the high arsenic water areas, and it was known that homocysteine could induce nerve cell damage. Thus, the homocysteine levels in rat serum and brain were measured. Data showed that rats in the 10 mg/L and 50 mg/L arsenite-treated group for 6 months had significantly higher homocysteine levels in serum and brain than in the control (*P* < 0.05 or *P* < 0.01). The serum homocysteine level in control was 5.73 ± 1.00 μmol/L and in arsenite-treated group for 6 months was 6.60 ± 0.98, 7.11 ± 1.12 and 6.78 ± 1.14 μmol/L, respectively. The brain homocysteine level in control was 12.27 ± 1.29 nmol/g and in arsenite-treated group for 6 months was 16.74 ± 2.58, 21.15 ± 4.95 and 21.28 ± 5.16 nmol/g, respectively. In the three groups of rats exposed to arsenite for 3 months and distilled water for another 3 months, the homocysteine in serum decreased significantly (5.06 ± 0.60, 5.69 ± 1.14 and 5.63 ± 0.97 μmol/L, respectively) than the corresponding 6 months groups (*P* < 0.05 or *P* < 0.01; Figure 4A). And the homocysteine in brain of rats in these three groups have shown a downward trend as compared with the corresponding 6 months groups. However, it did not have statistical difference because of a large variation among within group (Figure 4B). It may suggest that the homocysteine in brain was not stable after the arsenite was withdrawn. These findings indicate that arsenite could induce high homocysteine levels in serum and brain which could be partly recovered after withdrawal period.

### Morphological Changes of Hippocampus

**Figure 5:**
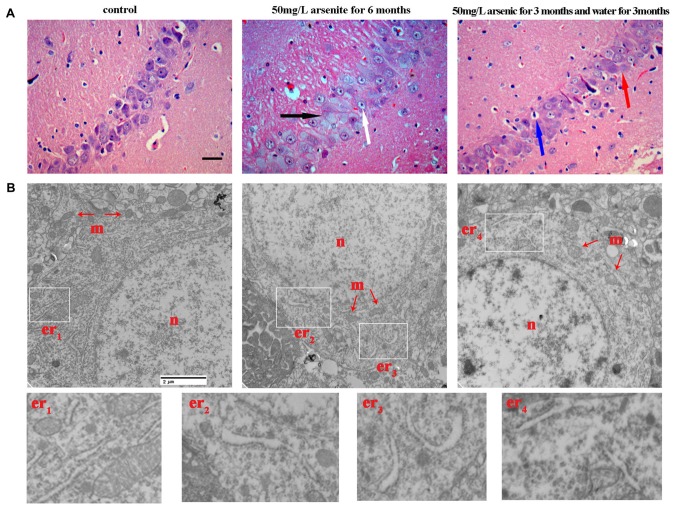
The morphological changes of hippocampus were observed with light microscope and transmission electronic microscope. **(A)** Representative images for histopathological changes in CA1 area of the hippocampus after HE staining (magnification 200×). Cells were arranged orderly and intact with nuclei stained clear in the control group. In 50 mg/L arsenite-treated for 6 months group, HE staining showed degenerative neurons (black arrow) and pyramidal cells became granular vacuolar changes and nuclear pyknosis (white arrow). In 50 mg/L arsenite-treated for 3 months and then distilled water for another 3 months group, neurons had better cell morphology than arsenite-treated for 6 months group, but it still could be observed mild vascular swelling (red arrow) and interstitial edema (blue arrow). Scale bar = 100 μm. **(B)** The ultrastructure of CA1 pyramidal neurons in the hippocampus were observed with electron microscope (magnification 20,000×). The changes in neuronal structures include the nucleus (n), mitochondria (m), and rough ER (er). Higher magnification of the white rectangular areas showed the changes of endoplasmic reticulum (ER). Scale bar = 2 μm.

Our MWM test suggests that arsenic exposure reduces the spatial learning and memory ability. The hippocampus, a part of the limbic system that is susceptible to excitotoxicity (Huo et al., 2015), is crucial to memory function and spatial navigation. The hippocampus has long been extensively studied for both its role in learning and memory (Mattfeld and Stark, 2015). So, we focused on the morphological changes of hippocampus induced by arsenite exposure. Based on pyramidal neuron morphology, the hippocampus can be divided into several regions, termed CA1–CA4. Through observing the changes of hippocampus by staining, we found that arsenite exposure could cause pathologic change in multiple regions of the hippocampus and the trend of changes was basically the same. According to the report, the function of pyramidal cells in the CA1 region were closely associated with long-term memory, the lack of CA1 pyramidal cells may even be sufficient to cause memory loss (Luo et al., 2015). Thus, to evaluate possible histopathological changes and ultrastructure changes in response to arsenite exposure, the CA1 region of hippocampus was studied by both conventional histological staining and TEM observation (Figure 5).

H&E staining revealed that, for the control group, cells were arranged orderly and intact with nuclei stained clear, dark blue. However, in 50 mg/L arsenite-treated for 6 months group, H&E staining showed more degenerative neurons and pyramidal cells underwent granular vacuolar changes and nuclear pyknosis. In 50 mg/L group arsenite-treated for 3 months and distilled water for another 3 months, neurons had better cell morphology than those in 50 mg/L arsenite-treated for 6 months, but mild vascular swelling and interstitial edema could still be observed (Figure 5A). The ultrastructural changes in neurons were observed with TEM (Figure 5B). In the control group, the nucleus had uniform chromatin and complete nuclear membrane. There were abundant organelles including rough ER, mitochondria, ribosomes and so on. In 50 mg/L arsenite-treated group for 6 months, the cells presented damage with the mitochondrial swelling and the loss of mitochondrial cristae. The lumen expansion of the ER, a morphological hallmark of ER stress, was observed. In the 50 mg/L group arsenite-treated for 3 months and distilled water for another 3 months, the lysosome increased and slight expansion of the ER was observed, which may suggest ER stress was mitigated by the arsenite withdrawn. The morphological changes of ER under TEM was a special discovery and a clue to study the ER stress in the next section.

### The Activation of PERK–eIF2α–ATF4 Pathway in the Hippocampus after Arsenite Exposure

**Figure 6:**
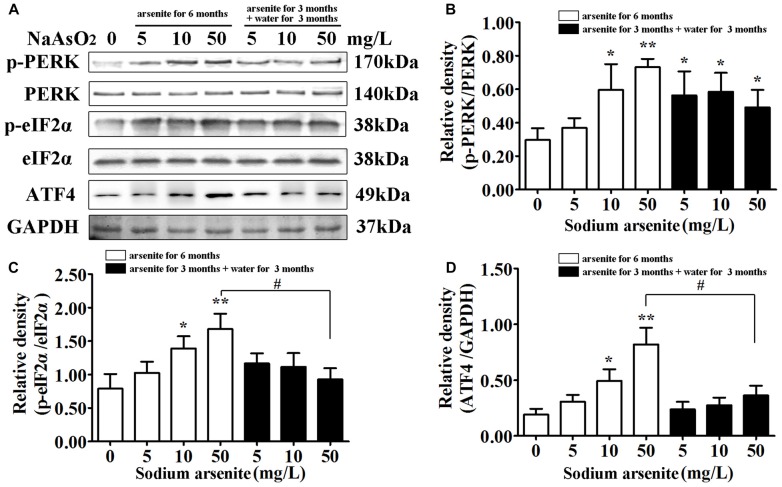
The activation of protein kinase-like ER kinase (PERK)–eIF2α–activating transcription factor 4 (ATF4) pathway in the hippocampus after arsenite exposure. **(A)** Immunoblot for p-PERK, total-PERK, p-eIF2α, total-eIF2α, ATF4 and GAPDH in the hippocampus. Relative density analysis of the p-PERK **(B)**, p-eIF2α **(C)** and ATF4 **(D)** protein bands. The relative density is expressed as the ratio (p-eIF2α/t-eIF2α; p-PERK/t-PERK; ATF4/GAPDH). Data were expressed as mean ± SD (*n* = 5). *P < 0.05, **P < 0.01 vs. control group. ^#^*P* < 0.05.

The underlying mechanisms from the perspective of ER stress were subsequently studied. The PERK signaling pathway is the initially activated pathway during ER stress. The phosphorylation of PERK and its downstream target eIF2α is a hallmark of activation of this pathway. The quantification of phospho-PERK and phospho-eIF2α band densities normalized to total PERK and total eIF2α revealed that arsenite induced increase of phospho-PERK and phospho-eIF2α (*P* < 0.05 or *P* < 0.01; Figures 6A–C), suggesting that PERK pathway is activated by arsenite exposure. The phosphorylation level of eIF2α was decreased significantly in the 50 mg/L group arsenite-treated for 3 months (then withdrawn for 3 months) than the level in the corresponding 6 months group (*P* < 0.05; Figures 6A,D). Based on these results, activation of the PERK pathway was induced by arsenite exposure.

### The Expression of GRP78 and CHOP in the Hippocampus after Arsenite Exposure

**Figure 7:**
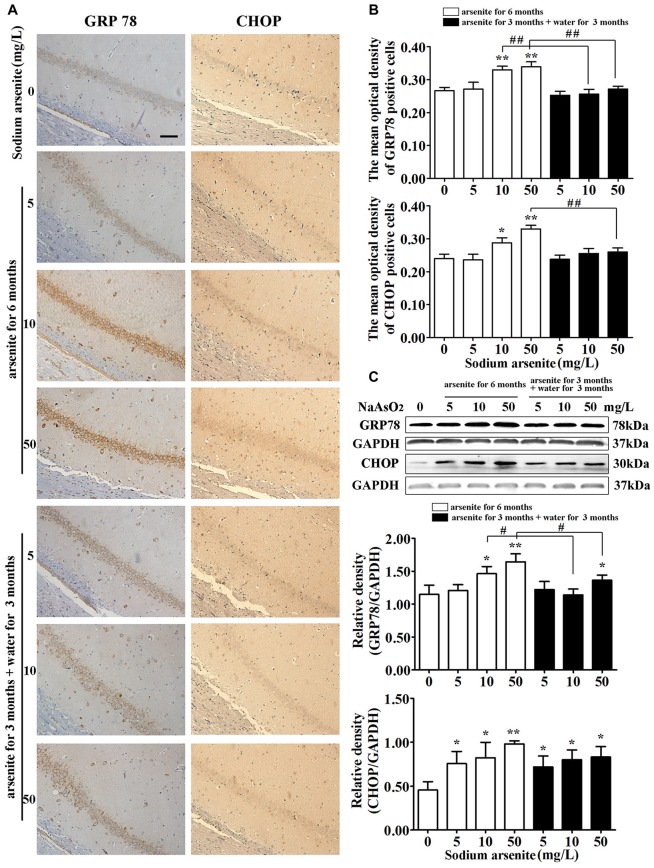
The expression of glucose-regulated protein 78 (GRP78) and CHOP in the hippocampus after arsenite exposure. **(A)** Representative photomicrographs of GRP78 and CHOP immunohistochemistry in the hippocampal CA1 sections respectively (magnification 200×). Scale bar = 100 μm. **(B)** Analysis of the mean optical density value of GRP78 and CHOP positive cells in the hippocampal CA1 sections. **(C)** Representative immunoblot for GRP78 and CHOP in the hippocampus and relative density analysis of the GRP78 and CHOP protein bands. The relative density is expressed as the ratio (GRP78/GAPDH, CHOP/GAPDH). Data were expressed as mean ± SD (immunohistochemistry, *n* = 8; immunoblot analysis, *n* = 5). *P < 0.05, **P < 0.01 vs. control group. ^#^*P* < 0.05, ^##^*P* < 0.01.

GRP78 is an ER resident molecular chaperone, so the expression of GRP78 in the hippocampus was detected by immunohistochemistry and immunoblot analysis. The results from immunohistochemistry revealed that the expression of GRP78 was increased significantly in CA1 region of the rat hippocampal in the 10 mg/L and 50 mg/L arsenite-treated groups for 6 months (*P* < 0.05 or *P* < 0.01), but the expression of GRP78 was decreased significantly in the 10 mg/L and 50 mg/L arsenite-treated groups for 3 months than the expression in the 10 mg/L and 50 mg/L groups arsenite-treated for 6 months, respectively (*P* < 0.01; Figures 7A,B). The results of immunoblot analysis also revealed that arsenite significantly increased the expression of GRP78 in a dose-dependent manner, and the expression of GRP78 was decreased significantly in the 10 mg/L and 50 mg/L groups arsenite-treated for 3 months (then withdrawn for 3 months) than the expression in the 10 mg/L and 50 mg/L arsenite-treated groups for 6 months, respectively (*P* < 0.05; Figure 7C). Meanwhile, the key UPR-proapoptotic player CHOP, that promotes transcription of proapoptotic genes, was detected by immunohistochemistry and immunoblot analysis. These results from both assays revealed that the expression of CHOP was increased significantly in CA1 region of the rat hippocampal in the 10 mg/L and 50 mg/L groups arsenite treated for 6 months (*P* < 0.05 or *P* < 0.01), but the expression of CHOP was decreased significantly in the 50 mg/L group arsenite-treated for 3 months than the expression in the 50 mg/L group arsenite-treated for 6 months (*P* < 0.01; Figures 7A–C).

### Correlation and Partial Correlation Analysis

Correlational analysis showed that arsenic in brain had significant positive correlation with homocysteine (*r* = 0.527, *P* = 0.001), GRP78 (*r* = 0.489, *P* = 0.003) and CHOP (*r* = 0.493, *P* = 0.003). Similarly, homocysteine was positively associated with GRP78 (*r* = 0.524, *P* = 0.001) and CHOP (*r* = 0.460, *p* = 0.005; Supplementary Table S1). Partial correlation analysis revealed that there were no significant correlations between arsenic in brain with the expression of GRP78 (*r* = 0.295, *P* = 0.091) or CHOP (*r* = 0.318, *P* = 0.066), after homocysteine was controlled (Supplementary Table S2). These results suggested that arsenite-induced ER stress might be related with the increasing of homocysteine under the arsenite exposure.

### Apoptosis Induced by ER Stress in the Hippocampus

**Figure 8:**
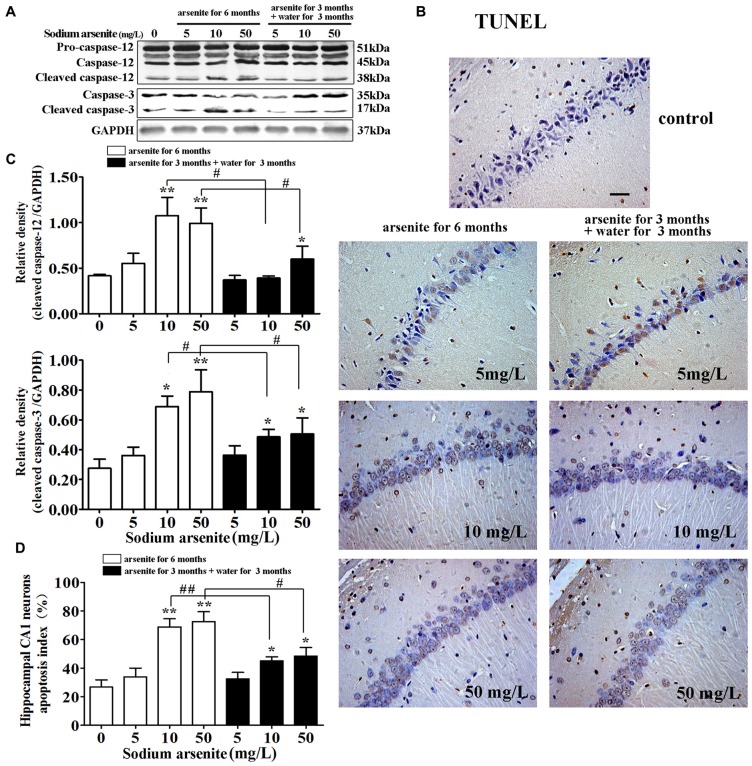
Apoptosis induced by ER stress in the hippocampus. **(A)** Representative immunoblot for cleaved caspase-12, cleaved caspase-3 and GAPDH. **(B)** Relative density analysis of the cleaved caspase-12 and cleaved caspase-3 protein bands. The relative density is expressed as the ratio (cleaved caspase-12/GAPDH; cleaved caspase-3/GAPDH; *n* = 5). **(C)** Photomicrographs of TUNEL-positive cells in the hippocampal CA1 of all treated groups (magnification 200×). Scale bar = 100 μm. **(D)** Hippocampal CA1 neurons apopotosis index in each group (*n* = 8). Data were expressed as mean ± SD. *P < 0.05, **P < 0.01 vs. control group. ^#^*P* < 0.05, ^##^*P* < 0.01.

It is known that excessive or continuous ER stress can lead to apoptosis by activating CHOP and caspase-12. Caspase-12 was specially expressed in the ER and the cleavage of caspase-12 were essential for ER stress-induced apoptosis (Szegezdi et al., 2003; Lai et al., 2007). So, the activation of caspase-12 was detected in this study. Immunoblot analysis showed that exposure of arsenite increased the expression of cleaved caspase-12 in the rat hippocampus (Figures 8A,B). Meanwhile, immunoblot analysis showed that exposure of arsenite increased the expression of cleaved caspase-3 in the rat hippocampus (Figures 8A,B). Conform to these results, the percentage of TUNEL-positive cells in CA1 region of the rat hippocampus in the 10 mg/L and 50 mg/L groups arsenite-treated for 6 months was significantly increased (*P* < 0.05 or *P* < 0.01; Figures 8B–D). Therefore, the arsenite stimulated cleavage of caspase-12 and enhanced apoptosis. However, in the 10 mg/L and 50 mg/L groups arsenite-treated for 3 months and distilled water for another 3 months, the expression of cleaved caspase-12 and cleaved caspase-3 decreased significantly and the percentage of TUNEL-positive cells also reduced in the hippocampus when compared with the corresponding 6 months group (*P* < 0.05 or *P* < 0.01; Figures 8A–D).

## Discussion

Previous animal studies have shown that brain arsenic levels have a dose-response relationship with levels of arsenic in drinking water, demonstrating that the BBB does not effectively block the passage of arsenic to the central nervous system (Rodríguez et al., 2002; Luo et al., 2009; Rai et al., 2010; Tolins et al., 2014). It is known that most arsenic existing in body will be excreted via urine. However, it is unclear whether the BBB can interfere with the excretion of arsenic from brain after withdrawal period. So, in this study experimental animals were divided into seven groups according to different arsenite exposure levels and time periods. In addition to the groups exposed to arsenite for 6 months, there were three groups exposed arsenite for 3 months and then received over to distilled water for another 3 months. Consistent with previous study, our results also indicated that arsenic could be absorbed and significantly accumulated in the blood and brain after exposure, and most arsenic can be excreted from the blood after withdrawal period. However, the brain arsenic levels still increased after the withdrawal period, suggesting that the excretion of arsenic from the brain may be limited by the BBB. Because age was an important factor that influenced the cognitive function, the animal experiment lasted for 6 months to eliminate the interference factor of age in this study.

In recent years, many studies have focused on neural injury after arsenic exposure including hippocampal function (Liu et al., 2012), increased Alzheimer’s-associated pathologies (Gharibzadeh and Hoseini, 2008; Gong and O’Bryant, 2010; Edwards et al., 2014), and adult neurogenesis (Tyler and Allan, 2013). These potential mechanisms are closely related with apoptosis of nerve cells. Apoptosis plays an important role in the development and progression of nerve functional disease. Recent studies have demonstrated that arsenic can induce neuron apoptosis through certain potential mechanisms, such as oxidative stress (Yen et al., 2011), stimulation of the N-methyl-D-aspartate (NMDA) receptor (Luo et al., 2009; Huo et al., 2015), synaptic remodeling (Luo et al., 2012), and stimulation of mitochondrial driven pathways (Flora et al., 2009; Dwivedi et al., 2011). However, the mechanism has not been fully clarified. In general, the MWM test is commonly used as a method of behavior assessment in the field of neurotoxicology (D’Hooge and De Deyn, 2001). In the MWM test, the performance of rats in a spatial navigation test reflects spatial learning ability, whereas the spatial probe test is a measure of spatial reference memory (Baldi et al., 2005). Previous studies have also reported that chronic arsenic exposure can negatively affect the spatial learning and memory of rats (Luo et al., 2009; Jing et al., 2012). Similarly, in our study, rats exposed to arsenite via drinking water also showed significant decreases in the learning and memory through the MWM test indicating that the rat cognitive damage model was successfully established.

These behavioral findings are mostly associated with hippocampal changes. In this study, pathological alterations (such as degenerative neurons, granular vacuolar changes and nuclear pyknosis) were observed in the hippocampus of arsenite-treated rats. Meanwhile, both the percentage of TUNEL-positive cells and of cleaved caspase-3 significantly increased in rats exposed to arsenite, suggesting that arsenite can induce neuronal apoptosis. It is worth noting that obvious lumen expansion of ER, a morphological hallmark of ER stress, was observed and was mitigated after 3 months of withdrawal. ER stress triggers UPR and plays important roles in apoptosis (Malhotra et al., 2008). Therefore, this result was the clue that arsenite could induce ER stress.

In this study, we demonstrated that ER stress may be related with apoptosis induced by arsenite. First, expression of ER-located chaperones GRP78 significantly increased in a dose-dependent manner in the hippocampus treated with arsenite, indicating that arsenic exposure induces ER stress. Second, the PERK-eIF2α-ATF4 pathway is activated by arsenite exposure. PERK is activated by dimerization and autophosphorylation which further phosphorylate and activate the translation initiation factor eIF2α (Pluquet et al., 2015). Our results revealed that arsenite induces the increase of phospho-PERK and phospho-eIF2α and increased the expression of ATF4, suggesting that PERK-eIF2α-ATF4 pathway is activated by arsenite exposure. Third, CHOP and cleaved caspase 12, the markers of ER stress mediated apoptosis were increased in the hippocampus exposed to arsenite. Excessive or prolonged ER stress may induce apoptosis, which is mediated by CHOP and caspase 12. The CHOP showed low expression in the physiological condition, but aggressive up-regulation under ER stress conditions (Chen et al., 2013; Li et al., 2015). In this study, immunoblot analysis and immunohistochemistry showed that CHOP expressed at very low levels in the control group, but was strongly activated in the groups treated with arsenite for 6 months. Caspase-12 was specifically located in the ER and played a critical role in ER stress-induced apoptosis (Hitomi et al., 2004). The activated CHOP subsequently leaded to activation and cleavage of caspase-12, which in turn triggered the caspase cascade, leading to cell death (Chen et al., 2013). Immunoblot analysis showed that arsenite exposure significantly increased the amount of cleaved caspase-12 in the rat hippocampus. These results suggested that sodium arsenite consumption via drinking water could lead to ER stress-induced apoptosis. Fourth, it was also supported by the finding that the expression of above mentioned factors which related with ER stress induced apoptosis were partially decreased after withdrawal period.

Epidemiological surveys suggested that homocysteine appears to be an important determinant of arsenic metabolism (Gamble et al., 2005; Hall et al., 2007). Homocysteine levels are closely associated with cognitive functions and high levels of homocysteine could induce apoptosis of neurons (Ganapathy et al., 2011). Previous studies have suggested that homocysteine induced oxidative stress by stimulating NMDA receptor-mediated neuronal nitric oxide synthase activation and by enhancing the production of free radicals, thereby causing a significant increases in neuronal apoptosis (Coimbra et al., 2014; Srejovic et al., 2015). Homocysteine also induces BBB disruption and synaptic remodeling in the hippocampus, in part by increasing neuronal damage (Kamat et al., 2016). Our previous experiment (rats were exposed to arsenite for 3 months and were sacrificed immediately) indicated that rats in the 10 mg/L and 50 mg/L groups arsenite-treated for 3 months showed significantly higher serum homocysteine levels than the control (*P* < 0.05; Supplementary Figure S1). In the present study, results of homocysteine levels in serum and in brain (arsenite treated for 6 months, or for 3 months and then followed by 3 months with distilled water) may indicate that homocysteine levels could be increased with the arsenite exposure and could be partly recovered by the change of arsenite to distilled water.

Certain studies have shown that homocysteine-induced ER stress has been implicated in the pathogenesis of central nervous system diseases (Hosoi et al., 2010). In order to discuss the relationships among arsenic exposure, the homocysteine levels and the marker protein of ER stress, correlation analysis and partial correlation analysis were conducted. The results suggested that arsenite induced ER stress was influenced by the enhancement of homocysteine. Despite all of this, results were only correlative and not necessarily causative. More intervention trials are needed, such as the treatment of ER stress inhibitors or homocysteine depletion *in vivo*, to confirm this mechanism.

The recommended maximum level of arsenic in drinking water in China is 0.05 mg/L. In some high arsenic exposed water areas such as Bangladesh, arsenic level in drinking water ranges from 0.25 to 2.1 mg/L (Chowdhury et al., 2000). In our preliminary experiments, the average daily water intake in 300–500 g rats was about 40 ml. Rats consumed 4.0–6.7 mg/kg sodium arsenite per day in the highest dose group (50 mg/L sodium arsenite). The Lethal Dose 50 (LD 50) of oral sodium arsenite in rats was 41 mg/kg. The highest dose is about one-tenth of the LD 50. This dosage of sodium arsenite were well tolerated by rats but could cause cognitive impairment by subchronic exposure (Sánchez-Peña et al., 2010; Ram Kumar et al., 2013). Rats have more endurance to arsenite than human beings. Although the doses of sodium arsenite used in this study was far above the human exposure levels in arsenic exposed areas, it might still be useful for inference to human exposure conditions.

**Figure 9:**
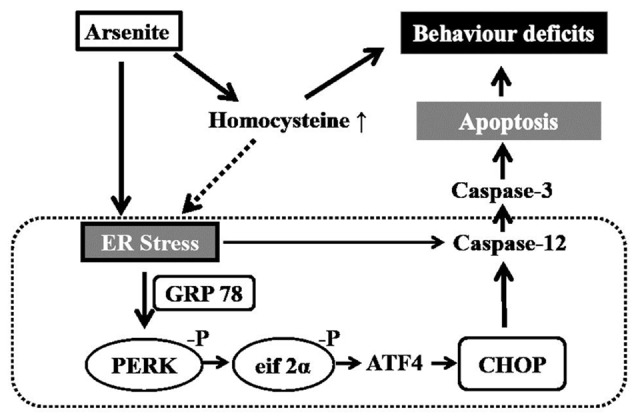
Schematic diagram of arsenite induced cognitive impairment in the hippocampus of rats.

In conclusion, the exposure to sodium arsenite via drinking water induced impairment of learning and memory ability. Potential mechanisms are related to enhancement of homocysteine and ER stress-induced apoptosis in the hippocampus shown in Figure 9.

## Author Contributions

DS and YG developed the research idea and experimental design. HNS performed experiments and participated in writing and editing of manuscript. YY provided essential advice to the project and participated in editing of manuscript. HWS contributed to the detection of homocysteine. WS contributed to the protein expression and purification. MG made the statistical analysis. HW, LJ and LQ helped to perform the animal studies.

## Conflict of Interest Statement

The authors declare that the research was conducted in the absence of any commercial or financial relationships that could be construed as a potential conflict of interest.

## Abbreviations

ATF4: Activating transcription factor 4
BBB: Blood-brain barrier
CHOP: C/EBP homologous protein
eIF2α: Eukaryotic translation initiation factor 2 subunit α
ER: Endoplasmic reticulum
GRP: Glucose-regulated protein
HE: Hematoxylin and eosin
PERK: RNA-regulated protein kinase-like ER kinase
UPR: Unfolded protein response.
