# Supplementary material for: Sodium Arsenite-Induced Learning and Memory Impairment Is Associated with Endoplasmic Reticulum Stress-Mediated Apoptosis in Rat Hippocampus
Source: Front Mol Neurosci. 2017 Sep 7;10:286. doi: 10.3389/fnmol.2017.00286 (PMC5594089; doi:10.3389/fnmol.2017.00286)
Supplement: Supplementary file 1 [file Data_Sheet_1.docx]

Supplementary Material

Sodium Arsenite Induced Learning and Memory Impairment through Inducing Apoptosis in Rat Hippocampus Mediated by Endoplasmic Reticulum Stress-Regulated Signaling Pathway

**Hongna Sun^1^, Yanmei Yang^1^, Hanwen Shao^1^, Weiwei Sun^1^, Muyu Gu^1^, Hui Wang^1^, Lixin Jiang^1^, Lisha Qu^1^, Dianjun Sun^1*^, Yanhui Gao^1,2*^**

*** Correspondence:** Dianjun Sun : [hrbmusdj@163.com](mailto:hrbmusdj@163.com) Yanhui Gao : [gaoyh411@163.com](mailto:gaoyh411@163.com)

1. **Supplementary Figure**

**
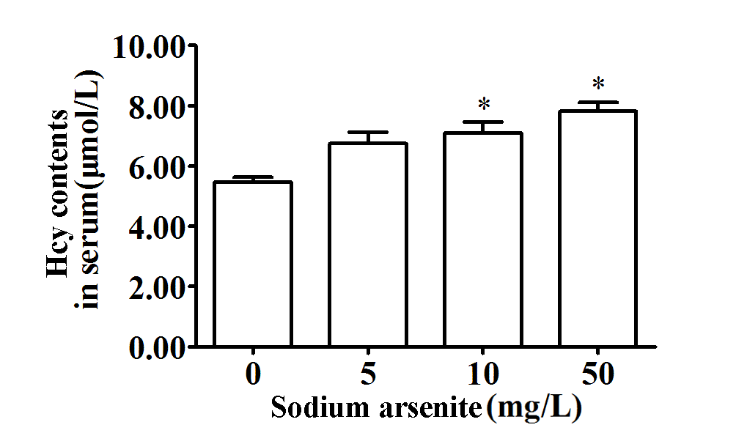
**

**Supplementary Figure 1.** The rats were exposed to arsenite (0, 5, 10, 50 mg/L) for 3 months and were sacrificed immediately. Homocysteine (Hcy) level in serum as shown in the figure. Results represent mean ± SD and are expressed in µmol homocysteine /L. n=8. *P < 0.05 versus control group.

1. **Supplementary Tables**

**Table 1** Correlation analysis among arsenic, homocysteine, GRP78, and CHOP in brain (n=35)

|  | arsenic (μg/g) | |  | homocysteine (nmol/g) | |
| --- | --- | --- | --- | --- | --- |
|  | *r* | *P* |  | *r* | *P* |
| homocysteine (nmol/g) | 0.527 | 0.001* |  |  |  |
| GRP78 | 0.489 | 0.003* |  | 0.524 | 0.001* |
| CHOP | 0.493 | 0.003* |  | 0.460 | 0.005* |

*P ＜0.01

**Table 2** Partial correlation analysis between arsenic and GRP78, CHOP, in brain (n=35)

|  | arsenic (μg/g) | |
| --- | --- | --- |
|  | *r* | *P* |
| GRP78 | 0.295 ^*^ | 0.091 |
| CHOP | 0.318 ^*^ | 0.066 |

*Homocysteine was controlled.
